# Sleep Related Breathing Disorders and Inflammation — The Missing Link? A Cohort Study Evaluating the Interaction of Inflammation and Sleep Related Breathing Disorders and Effects of Treatment

**DOI:** 10.1371/journal.pone.0137594

**Published:** 2015-09-10

**Authors:** Natascha Troester, Michael Palfner, Erich Schmidberger, Horst Olschewski, Alexander Avian

**Affiliations:** 1 Division of Pulmonology, Department of Internal Medicine, Medical University, Graz, Austria; 2 Institute for Medical Informatics, Statistics and Documentation, Medical University, Graz, Austria; 3 Ludwig Boltzmann Institute for Lung Vascular Research, Graz, Austria; University Heart Center Freiburg, GERMANY

## Abstract

**Introduction:**

Sleep related breathing disorders (SRBD) are associated with both obesity and systemic inflammation. While the relationship between obesity and SRBD is established, the causality between inflammation and SRBD remains unclear. In this study we investigated the relation between SRBD and C-reactive protein (CRP) as a parameter of inflammation and the influence of SRBD treatment on CRP with additional regard to changes in metabolic and cardiovascular parameters.

**Methods:**

Polysomnography (PSG) and laboratory data of patients diagnosed with SRBD over a period of 5 years were prospectively collected in a database and retrospectively analysed regarding the association of SRBD (according to apnoea-hypopnoea- index (AHI), duration of events and extent of desaturation) to CRP, blood pressure, cholesterol, fasting plasma glucose, HbA1c, quality of life measured via a visual analogue scale (VAS 0–100%), and the effects of SRBD therapy on these parameters.

**Results:**

716 patients were included in the study, 171 with mild SRBD (AHI ≥5 to <15/h), 209 with moderate SRBD (AHI 15 to <30/h), 336 with severe SRBD (AHI ≥30/h). **Results according to severity of SRBD**. Severe SRBD was significantly associated with elevated levels of CRP (3.7 [1.8–7.0] mg/l, vs. moderate (p = 0.001), and mild SRBD (p<0.001), and higher prevalence of hypertension as compared to moderate and mild SRBD (p<0.001, respectively). **Results in highly successful treatment**. If SRBD treatment was highly successful (AHI <5/h), CRP and quality of life improved significantly (p = 0.001 and p = 0.002), as did blood pressure (p<0.001 for systolic and diastolic values), although BMI increased (p<0.001). **Results in partially successful treatment**. If success was defined as reduction of AHI of ≥50%, CRP also decreased (p<0.001), as did blood pressure (p<0.001). Again, BMI increased (p<0.001).

**Conclusion:**

This is the first study to show an association of SRBD and CRP independently of BMI in a large cohort. The SRBD therapy-induced CRP decrease was not associated with BMI changes or metabolic changes but rather with the magnitude of AHI improvement.

## Introduction

Sleep related breathing disorders (SRBD) comprise various types. The most important and predominant ones are obstructive sleep apnoea (OSAS) and central sleep apnoea (CSAS), although with distinct differences in pathophysiology. In OSAS, repeated episodes of upper airway closure (leading to cessation of respiratory flow = apnoea, or diminished flow with hypoxia and/or arousal = hypopnoea []) during sleep lead to intermittent hypoxia, sympathetic activation, cytokine release and cardiovascular morbidity and mortality [1]. The estimated prevalence is approximately 20 to 30 percent in males and 10 to 15 percent in females when OSAS is defined as an AHI >5 events per hour as measured by a polysomnography [2].

In CSAS, episodes of apnoea result from temporary suspension of ventilatory effort. This is due to disturbances in ventilatory response which is dependent on metabolic control system (chemoreceptors, pCO_2_). This type includes idiopathic central sleep apnoea as well as secondary CSAS such as Cheyne-Stokes respiration, hypnotics- and high-altitude induced CSAS [3]. Effects on morbidity and mortality may be similar to OSAS, but fewer data exist according to apparently lower prevalence.

Sleep-related breathing disorders (SRBD) are associated with increased cardiovascular morbidity and mortality [4, 5, 6]. Cardiovascular disease is associated with systemic inflammation [7, 8, 9, 10, 11, 12], and this may be the link between SRBD and cardiovascular disease [13, 14, 15]. Accordingly, elevated C-reactive protein (CRP) plasma levels were found in SRBD [16, 17, 18, 19], and nasal continuous positive airway pressure (CPAP) not only improved SRBD but also CRP levels [20, 21, 22]. However, obesity which is strongly associated with SRBD, may also be associated with inflammation [23, 24, 25] posing the question if obesity is the true link between SRBD and inflammation. The aim of this study was to analyse CRP in association with SRBD in different levels of severity as well as changes of CRP, BMI, BP, and QoL with SRBD therapy.

## Methods

We reviewed the data of all patients referred to the sleep laboratory of the Department of Internal Medicine/Division of Pulmonology at the Medical University of Graz, Austria, between 2007 and 2012. Data had been prospectively entered into a database. All patients diagnosed with SRBD and at least one polysomnographic control after established positive airway pressure or positional therapy (at least three months after initiation) were included. The study was approved by the local ethics committee at the Medical University of Graz. Patients´ written consent was obtained.

PSG examination was conducted using a standard montage according to AASM [26]. Polysomnograms were scored by a polysomnographic technologist and examined by a sleep specialist. Polysomnography was performed using a digital device (Schwarzer™, Domino™). Respiratory events and sleep staging were scored according to the rules of the AASM [26].

Treatment consisted of CPAP, AutoCPAP, or bilevel positive airway pressure (BPAP), depending on patient characteristics or patient needs. In case of central sleep apnoea or complex sleep apnoea, adaptive servoventilation (ASV) was applied if necessary. All currently available types of CPAP/AutoCPAP/BPAP/ASV devices by Philipps Respironics® Inc., ResMed® Inc., and Weinmann® were used. Only 18 patients used positional therapy as long-term treatment. Titration was performed by a nurse specialist in sleep medicine according to instructions by the sleep specialist physician.

The evaluated parameters were apnoea-hypopnoea index (AHI), longest duration of a respiratory event (longest event), minimum capillary oxygen saturation level during sleep (minSpO_2_), oxygen desaturation index (ODI), percent of total sleep time below 90% SpO_2_ (%TST<90). Additional measurements included weight, height, body mass index (BMI), and clinical chemistry from blood samples taken in the morning after the overnight polysomnography. The evaluated parameters were C-reactive protein (CRP), high-density lipoprotein cholesterol (HDL), low-density lipoprotein cholesterol (LDL), total cholesterol (Chol), triglycerides (TG), fasting plasma glucose (FPG), and glycosylated haemoglobin (HbA1c). The following co-morbidities were evaluated: coronary artery disease (CAD), atrial fibrillation (AF), apoplexy (APO), arterial blood pressure (BP), renal insufficiency (GFR according to MDRD). All patients were asked to estimate their overall quality of life (Qol) on a visual analogue scale (VAS, 0% = worst possible, 100% = best possible) before and after treatment.

All data were stored and processed in a central database that automatically extracted data from the sleep laboratory system and was fed manually for the resting parameters. The patients were divided into SRBD patients (AASM category II, Sleep-Related Breathing Disorders) and others (AASM category I, III-VIII). Overall and in each subgroup changes in parameters from baseline to the final examination were evaluated by Wilcoxon- signed-rank test. For continuous variables differences between groups were analysed using Kruskal-Wallis Test. To analyse differences in categorical variables Chi square Test and, when appropriate, Fisher’s exact Test were used. To analyse associations between continuous variables Spearman’s rank correlation (ρ) was calculated. Statistical analyses were performed using SPSS 19.0 (SPSS, Chicago, IL, USA). Continuous data are presented as median and interquartile range. Categorical data are presented as absolute and relative frequencies.

## Results

From 2007 to 2012, 716 patients who underwent diagnostic PSG revealing SRBD started SRBD therapy and had at least one PSG control within this period (m/f: 512/204, 71.5%/28.5%). An initial AHI of ≤5 to <15/h (mild SRBD), of 15 to <30/h (moderate SRBD), and of ≥30/h (severe SRBD) was found in 171 (m/f: 117/54, 68%/32%), 209 (m/f: 139/70, 67%/33%), and 336 (m/f: 256/80, 76%/24%) patients. Characteristics at the diagnostic and final PSG as well as changes are shown in Tables 1–3, results according to success in Tables 4 and 5, comparison between the severity groups in Table 6.

**Table 1:** Characteristics of patients with mild sleep apnoea syndrome; median (interquartile range).

preAHI > = 5 bis <15		initial PSG	final PSG	Change	sign. change
Age	years	58.1 (50.2–69.2)	59.5 (51.1–70.1)	1.1 (0.5–1.7)	<.001
Height	cm	171.0 (164.0–176.0)	171.0 (164.0–176.0)	0.0 (0.0–0.0)	n/a
Weight	kg	86.0 (74.0–96.0)	87.0 (77.0–98.0)	0.0 (-1.0–3.0)	.035
BMI	kg/m²	29.3 (26.5–33.0)	29.4 (26.8–33.8)	0.0 (-0.4–1.1)	.035
HDL	mg/dl	53.0 (42.0–63.0)	51.0 (41.0–64.0)	0.0 (-6.0–5.0)	.803
LDL	mg/dl	111.0 (92.0–139.0)	114.5 (85.5–135.0)	-1.0 (-12.0–10.0)	.564
Chol	mg/dl	189.0 (162.0–220.0)	182.5 (156.0–216.0)	-5.0 (-21.0–12.0)	.044
CRP	mg/l	2.0 (1.0–4.4)	1.9 (1.0–3.8)	0.0 (-1.0–0.6)	.296
TG	mg/dl	114.0 (92.0–173.0)	116.0 (88.0–163.0)	-3.0 (-27.0–16.5)	.105
FPG	mg/dl	97.0 (89.0–109.0)	99.0 (91.0–112.0)	2.0 (-5.0–11.0)	.013
HbA1c	IFCC: mmol/mol	37.7 (35.5–42.1)	37.7 (35.5–42.1)	0.9 (-1.1–2.2)	.026
AHI		10.7 (8.6–12.8)	1.6 (0.4–4.0)	-8.5 (-10.7–-5.5)	<.001
Longest event	sec	55.8 (43.0–80.5)	31.0 (20.0–48.0)	-22.7 (-46.5–-4.2)	<.001
Min O2	%	82.9 (78.9–86.9)	89.0 (86.0–91.0)	6.0 (2.1–9.1)	<.001
ODI		9.7 (7.3–12.4)	1.5 (0.3–4.5)	-7.1 (-9.8–-4.3)	<.001
% TST<90		2.7 (0.6–13.3)	0.0 (0.0–1.2)	-1.9 (-10.7–-0.3)	<.001
BP syst	mmHg	137.5 (125.0–151.0)	134.0 (123.0–146.0)	-2.0 (-15.5–8.0)	.010
BP diast	mmHg	89.0 (81.0–97.0)	85.0 (77.0–92.0)	-3.0 (-11.0–3.0)	<.001
QoL		72.5 (53.8–80.0)	80.0 (60.0–90.0)	0.0 (-5.0–20.0)	<.001

**Table 2:** Characteristics of patients with moderate sleep apnoea syndrome; median (interquartile range).

preAHI 15 bis < 30		initial PSG	final PSG	Change	Significance of change
Age	years	63.0 (54.4–69.9)	64.4 (55.8–71.5)	1.1 (0.6–2.0)	<.001
Height	cm	170.0 (163.0–176.0)	170.0 (163.0–176.0)	0.0 (0.0–0.0)	n/a
Weight	kg	87.0 (78.5–99.0)	89.5 (79.0–100.0)	1.0 (-1.0–4.0)	<.001
BMI	kg/m²	30.3 (27.4–34.1)	30.8 (27.5–34.6)	0.4 (-0.4–1.3)	<.001
HDL	mg/dl	50.0 (41.0–61.0)	50.0 (43.0–61.0)	0.0 (-5.0–6.0)	.779
LDL	mg/dl	114.0 (92.0–140.0)	115.0 (95.0–142.0)	3.0 (-20.0–18.0)	.784
Chol	mg/dl	192.0 (163.0–220.5)	195.0 (167.0–223.0)	0.0 (-17.0–20.0)	.385
CRP	mg/l	2.5 (1.3–5.1)	2.3 (1.1–4.8)	-0.2 (-1.1–0.7)	.160
TG	mg/dl	124.0 (91.0–189.0)	128.0 (84.0–187.0)	3.0 (-24.5–30.5)	.201
FPG	mg/dl	100.0 (93.0–115.5)	100.0 (95.0–115.0)	1.0 (-7.0–9.0)	.197
HbA1c	IFCC: mmol/mol	37.7 (35.5–41.0)	38.8 (35.5–42.1)	0.2 (-1.1–2.2)	.006
AHI		22.1 (17.8–25.8)	2.0 (0.8–5.1)	-18.3 (-22.7–-14.8)	<.001
Longest event	sec	63.0 (45.0–93.0)	37.0 (24.6–56.9)	-26.0 (-51.0–-2.2)	<.001
Min O2	%	81.0 (76.0–84.9)	89.0 (86.9–91.0)	7.1 (4.0–11.1)	<.001
ODI		21.1 (17.1–25.9)	1.8 (0.5–5.7)	-17.5 (-22.3–-12.8)	<.001
% TST<90		5.8 (2.0–19.4)	0.0 (0.0–0.8)	-4.3 (-13.2–-1.1)	<.001
BP syst	mmHg	141.0 (129.0–154.0)	133.0 (126.0–149.0)	-3.5 (-15.0–9.0)	.011
BP diast	mmHg	88.0 (79.0–95.0)	84.0 (77.0–92.0)	-3.0 (-12.0–5.0)	<.001
QoL		70.0 (50.0–90.0)	80.0 (60.0–90.0)	0.0 (0.0–15.0)	.001

**Table 3:** Characteristics of patients with severe sleep apnoea syndrome; median (interquartile range).

preAHI > = 30		initial PSG	final PSG	Change	sign. change
Age	years	61.8 (51.2–69.9)	63.2 (51.7–71.5)	1.1 (0.6–1.8)	<.001
Height	cm	170.0 (164.0–177.0)	170.0 (164.0–177.0)	0.0 (0.0–0.0)	n/a
Weight	kg	98.0 (85.0–112.0)	97.0 (85.0–113.0)	0.0 (-2.0–2.5)	.402
BMI	kg/m²	34.6 (30.2–38.8)	34.5 (30.1–38.4)	0.0 (-0.7–0.9)	.391
HDL	mg/dl	48.0 (39.0–59.0)	49.0 (40.0–58.0)	-1.0 (-5.0–5.0)	.418
LDL	mg/dl	112.0 (92.0–131.5)	109.0 (89.0–132.0)	0.0 (-17.0–14.0)	.566
Chol	mg/dl	187.0 (159.0–214.0)	183.5 (156.0–213.0)	-1.0 (-18.0–15.0)	.372
CRP	mg/l	3.7 (1.8–7.0)	3.0 (1.6–5.8)	-0.4 (-2.2–0.8)	<.001
TG	mg/dl	132.0 (98.0–179.0)	131.5 (96.0–184.0)	1.0 (-28.0–30.0)	.676
FPG	mg/dl	104.0 (96.0–120.0)	105.0 (97.0–120.0)	1.0 (-7.0–8.0)	.624
HbA1c	IFCC: mmol/mol	39.9 (36.6–45.4)	40.0 (36.6–45.4)	0.0 (-2.2–2.2)	.238
AHI		49.9 (38.4–64.3)	2.9 (0.9–6.7)	-43.7 (-58.7–-33.2)	<.001
Longest event	sec	68.0 (51.1–92.0)	41.0 (25.8–70.0)	-24.1 (-51.1–0.8)	<.001
Min O2	%	75.9 (66.0–80.0)	88.0 (85.0–90.0)	12.1 (7.0–21.0)	<.001
ODI		50.3 (37.2–63.8)	3.0 (0.8–7.7)	-43.1 (-58.3–-32.9)	<.001
% TST<90		27.2 (11.5–52.3)	0.2 (0.0–2.0)	-23.7 (-48.4–-10.1)	<.001
BP syst	mmHg	143.0 (131.0–157.0)	137.0 (125.0–152.5)	-5.0 (-18.5–7.0)	<.001
BP diast	mmHg	90.0 (81.5–99.0)	85.0 (77.0–93.0)	-6.0 (-15.0–4.5)	<.001
QoL		75.0 (56.3–80.0)	80.0 (60.0–90.0)	0.0 (-10.0–20.0)	.004

**Table 4:** Characteristics of the success group; median (interquartile range).

postAHI < 5/h		initial PSG	final PSG	Change	sign. change
Age	years	60.2 (51.7–69.6)	61.7 (52.7–70.9)	1.1 (0.6–1.8)	<.001
Height	cm	170.0 (164.0–176.0)	170.0 (164.0–176.0)	0.0 (0.0–0.0)	n/a
Weight	kg	91.0 (80.0–106.0)	92.0 (81.0–105.0)	1.0 (-1.0–3.0)	<.001
BMI	kg/m²	31.6 (27.7–35.6)	31.7 (28.0–36.3)	0.3 (-0.5–1.1)	.000
HDL	mg/dl	50.0 (41.0–61.0)	50.0 (41.0–61.0)	0.0 (-5.0–5.0)	.716
LDL	mg/dl	114.0 (92.0–138.0)	114.0 (90.0–137.0)	-1.0 (-15.0–15.0)	.806
Chol	mg/dl	190.0 (161.0–220.0)	189.0 (161.0–218.0)	-2.0 (-18.0–17.0)	.409
CRP	mg/l	2.6 (1.2–5.5)	2.3 (1.1–4.6)	-0.2 (-1.5–0.6)	.001
TG	mg/dl	123.5 (94.5–174.5)	126.0 (90.0–173.0)	1.0 (-27.0–24.0)	.900
FPG	mg/dl	100.0 (92.0–113.0)	102.0 (94.0–115.0)	1.0 (-6.0–10.0)	.003
HbA1c	IFCC: mmol/mol	38.8 (35.5–43.2)	38.8 (35.5–43.2)	0.0 (-1.9–2.2)	.190
AHI		26.5 (14.5–46.0)	1.2 (0.4–2.5)	-25.0 (-44.1–-12.6)	<.001
Longest event	sec	64.5 (47.7–90.0)	30.0 (19.2–48.1)	-30.0 (-57.7–-11.0)	<.001
Min O2	%	79.9 (72.9–83.9)	90.0 (87.0–91.0)	9.1 (6.0–15.0)	<.001
ODI		25.6 (12.9–45.0)	1.2 (0.3–2.7)	-23.0 (-43.3–-10.9)	<.001
% TST<90		11.1 (2.6–37.1)	0.0 (0.0–0.4)	-9.8 (-31.5–-1.9)	<.001
BP syst	mmHg	141.0 (130.0–155.0)	135.0 (125.0–150.0)	-5.0 (-17.0–8.0)	<.001
BP diast	mmHg	90.0 (81.0–97.0)	85.0 (78.0–92.0)	-4.0 (-12.0–4.0)	<.001
QoL		75.0 (60.0–80.0)	80.0 (70.0–90.0)	0.0 (-5.0–20.0)	<.001

**Table 5:** Characteristics of the 50%success group; median (interquartile range).

Success—50%	initial PSG	final PSG	Change	sign. change
Age	60.6 (52.2–69.7)	62.1 (53.2–71.1)	1.1 (0.6–1.9)	<.001
Height	170.0 (164.0–176.0)	170.0 (164.0–176.0)	0.0 (0.0–0.0)	n/a
Weight	93.0 (81.0–106.0)	93.0 (82.0–106.0)	0.0 (-2.0–3.0)	<.001
BMI	32.0 (28.0–36.2)	32.2 (28.4–36.8)	0.0 (-0.6–1.1)	<.001
HDL	49.0 (40.0–60.0)	50.0 (41.0–60.0)	0.0 (-5.0–5.0)	.788
LDL	113.0 (92.0–136.0)	112.0 (90.0–136.0)	0.0 (-16.0–14.0)	.467
Chol	189.0 (160.5–218.0)	187.0 (159.0–217.0)	-2.0 (-18.0–16.0)	.228
CRP	2.9 (1.3–5.7)	2.5 (1.2–4.9)	-0.2 (-1.5–0.7)	<.001
TG	125.5 (96.0–179.0)	128.0 (91.0–181.0)	1.0 (-27.0–28.0)	.601
FPG	101.0 (93.0–116.0)	102.0 (95.0–116.0)	1.0 (-7.0–9.0)	.025
HbA1c	38.8 (35.5–43.2)	39.0 (35.5–44.0)	0.0 (-2.1–2.2)	.184
AHI	29.9 (16.6–49.9)	1.8 (0.6–4.2)	-26.6 (-45.1–-14.2)	<.001
Longest event	64.7 (47.5–90.8)	35.0 (23.0–57.0)	-27.0 (-52.3–-3.8)	<.001
Min O2	79.0 (71.9–83.9)	89.0 (86.0–91.0)	9.1 (5.1–15.1)	<.001
ODI	28.8 (15.6–50.3)	1.8 (0.5–5.0)	-25.2 (-43.3–-12.6)	<.001
% TST<90	13.2 (2.9–41.3)	0.1 (0.0–0.9)	-11.9 (-36.8–-2.5)	<.001
BP syst	141.5 (130.0–155.0)	135.0 (125.0–151.0)	-4.0 (-16.0–8.0)	<.001
BP diast	90.0 (81.0–98.0)	85.0 (77.0–93.0)	-4.0 (-13.0–4.0)	<.001
QoL	75.0 (60.0–80.0)	80.0 (60.0–90.0)	0.0 (-5.0–20.0)	<.001

**Table 6:** Comparison between different levels of severity of sleep apnoea syndrome; p-values.

		Mild vs moderate	Severe vs mild	Severe vs moderate
Age	years	0.016	0.385	0.435
Height	cm	0.834	1.000	0.868
Weight	kg	0.662	0.000	0.000
BMI	kg/m²	0.108	0.000	0.000
HDL	mg/dl	1.000	0.044	0.144
LDL	mg/dl	1.000	1.000	0.707
Chol	mg/dl	1.000	1.000	0.444
CRP	mg/l	0.153	0.000	0.001
TG	mg/dl	1.000	0.088	0.556
FPG	mg/dl	0.056	0.000	0.009
HbA1c	IFCC: mmol/mol	1.000	0.000	0.000
AHI		0.000	0.000	0.000
Longest event	sec	0.077	0.000	0.397
Min O2	%	0.011	0.000	0.000
ODI		0.000	0.000	0.000
% TST<90		0.002	0.000	0.000
BP syst	mmHg	0.107	0.003	0.190
BP diast	mmHg	0.476	0.235	0.044

### CRP, BMI and metabolic parameters according to SRBD severity

In severe SRBD, CRP showed the highest values (3.7 [1.8–7.0] mg/l) in comparison to moderate (2.5 [1.3–5.1] mg/l, p = 0.001) and mild SRBD (2.0 [1.0–4.4] mg/l, p<0.001). Severe SRBD patients also had the highest BMI (34.6 [30.2–38.8] kg/m^2^) in comparison to moderate (30.3 [27.4–34.1] kg/m^2^, p<0.001) and mild SRBD (29.3 [26.5–33.0] kg/m^2^, p<0.001). TG, FPG, HbA1c increase was also associated with SRBD severity, whereas Chol and LDL values did not show a significant association to severity.

### Cardiovascular events and co-morbidities according to SRBD severity

Systemic arterial hypertension (treated or untreated) was significantly more often present in severe SRBD (72%, 80%, and 86% in mild, moderate, and severe SRBD, p<0.001). The number of cardiovascular events and the extent of co-morbidities were slightly higher in severe SRBD, but these differences between groups were not statistically significant. Atrial fibrillation was present in 9%, 9%, and 11% in mild, moderate and severe SRBD (p = 0.625). Coronary artery disease was present in 13%, 14%, and 18% in mild, moderate, and severe SRBD (p = 0.220). A history of myocardial infarction was present in 5%, 8%, and 10% in mild, moderate, and severe SRBD (p = 0.124). Apoplexy was present in 5%, 7%, and 7% in mild, moderate, and severe SRBD (p = 0.507). GFR according to MDRD showed a normal renal function in 25%, 24%, and 22% in mild, moderate, and severe SRBD (p = 0.696).

### Results within groups of SRBD severity

SRBD therapy improved polysomnographic values in all groups. Non-satisfactory results in a few patients were due to final PSG without PAP due to lack of tolerance or due to on-going adaptation of treatment. Metabolic and inflammatory parameters showed variable changes (see Tables 1, 2 and 3):

CRP decreased significantly with therapy only in severe SRBD (p<0.001) who had also the highest baseline levels, while the changes in mild and moderate SRBD were not significant.

Systemic arterial blood pressure (BP) decreased in all groups (mild SRBD: systolic and diastolic p = 0.010 and diastolic p<0.001, moderate SRBD: p = 0.011 and p = 0.001, severe SRBD: p<0.001 and p<0.001). The same was true for quality of life (VAS 1–100) which improved in mild, moderate, and severe SRBD (72.5 to 80, p<0.001, 70 to 80, p = 0.001, 75 to 80, p = 0.004).

Patients with mild and moderate SRBD revealed a significant BMI increase during SRBD therapy (mild SRBD p = 0.032, moderate SRBD p<0.001), while in the severe SRBD group BMI was stable (p = 0.391). Accordingly, HbA1c increased in both mild (p = 0.026) and moderate SRBD (p = 0.006), no significant changes occurred in severe SRBD (p = 0.238). Fasting plasma glucose increased significantly in mild SRBD (p = 0.013), but not in moderate SRBD (p = 0.197) or in severe SRBD (p = 0.624). In contrast, total cholesterol decreased in mild SRBD (p = 0.044), while changes in moderate and severe SRBD were not significant. Triglycerides did not change during treatment in any group, neither did LDL (see Tables 1, 2 and 3).

### Effects of normalization of AHI after SRBD therapy

**Fig 1:**
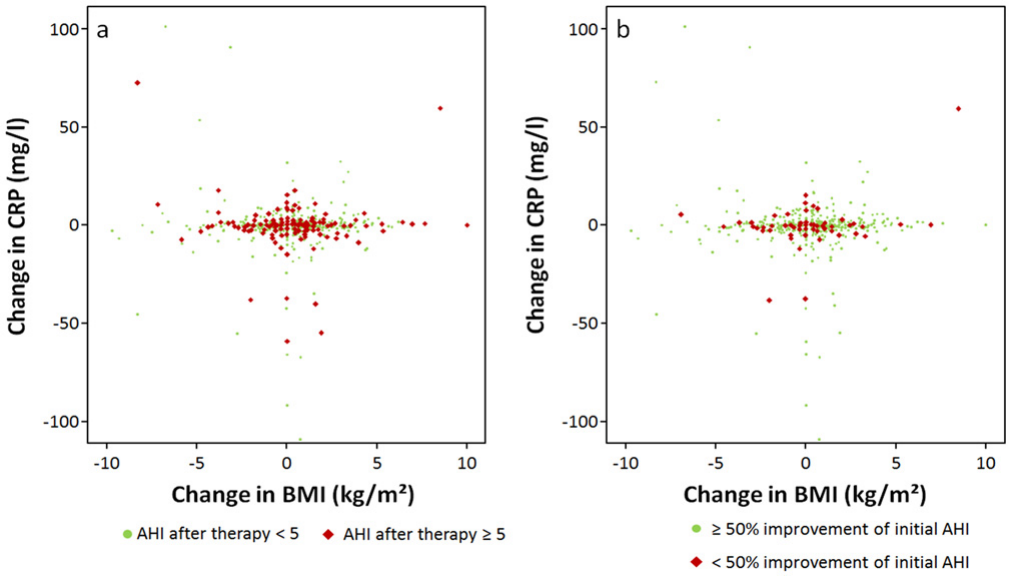
Correlation of changes of BMI and CRP: Change of BMI is not associated with change in CRP. **a** Success group (AHI<5/h) vs non-success. **b** 50%success group vs non-success.

**Fig 2:**
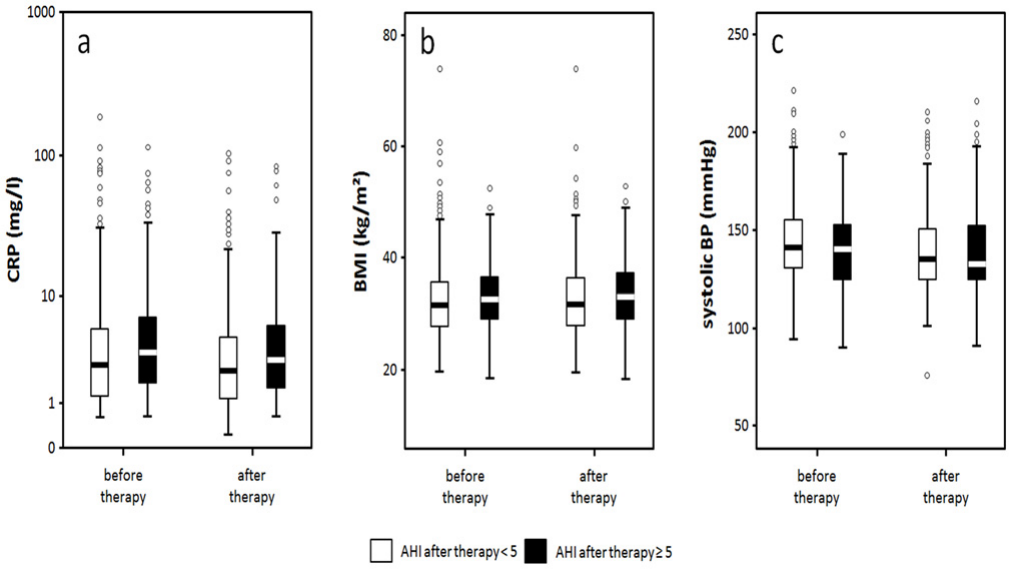
Development of CRP (a), BMI (b) and systolic blood pressure (c) in success (AHI<5/h) vs non-success.

When patients with satisfactory results (AHI <5/h) after sleep apnoea therapy were compared to patient with non-satisfactory results (AHI ≥5/h) after SRBD therapy, there were distinct differences with respect to changes in weight and laboratory values (Table 4, Table A and Table B in S1 File):

516 patients were assigned to the successful treatment group (initially mild SRBD n = 139, moderate SRBD n = 154, severe SRBD n = 223), 200 were assigned to the non-success group (mild SRBD n = 32, moderate SRBD n = 55, severe SRBD n = 113), although their PSG parameters improved significantly. Distribution of male and female patients in both groups were comparable (p = 0.582, success: f: 74%, m: 71%). After therapy, CRP decreased and changes were independent of BMI (Fig 1a). Decrease of CRP was significant in both treatment groups (p = 0.001, p = 0.047, Fig 2a) with greater changes in the success group (Table B in S1 File, p = 0.808). In contrast to CRP, BMI increased significantly in the success group (p<0.001, Fig 2b). BP improved in the success group (p<0.001 for systolic and diastolic values) and in the non-success group (p = 0.036 systolic, p<0.001 diastolic) within similar ranges (p = 0.184 systolic, p = 0.403 diastolic, Fig 2c).

HbA1c and FPG showed no significant improvement or even worsening. Quality of life was significantly higher in the success group than in the non-success group (VAS = 80 vs 73, p = 0.002).

The period of time between initial and final examination was not the same for all patients. Patients with finally lower AHI had a longer period of time between the initial and the final examination (final AHI<5/h: 12.5 months [6.7–22.2], final AHI ≥5/h: 11.3 months [5.2–23.6], p = 0.036), but this yielded no correlation with CRP (AHI<5/h: ρ = -0.026; p = 0.560, AHI ≥5/h: ρ = 0.028; p = 0.692).

Overall correlation of time period to CRP did not yield statistically significant results, either (ρ = -0.005, p = 0.897).

### Effects of 50% improvement of AHI

**Fig 3:**
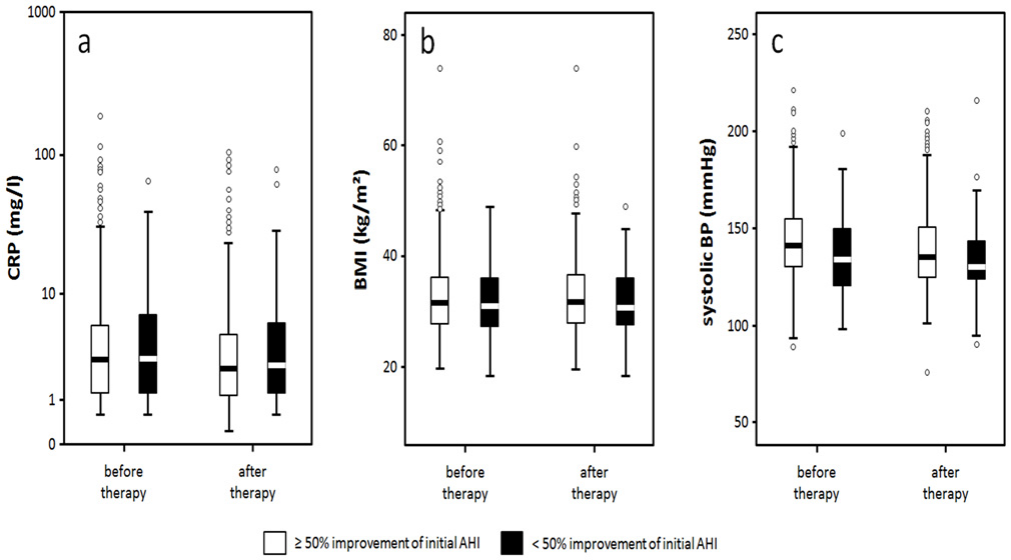
Development of CRP (a), BMI (b) and systolic blood pressure (c) in 50%success vs non-success.

If treatment success was defined as improvement by at least 50% of initial AHI (success50%), 644 patients were in the success group, and 69 in the non-success group with no statistically significant difference regarding gender distribution (p = 0.346, success: f 89%, m 91%). The success group turned out to be younger, with initially more severe SRBD according to AHI, ODI, longest event, O2 saturation and had higher values of BP (see Table 5, and Table C and Table D in S1 File). As expected, the success group improved more distinctly in AHI, ODI, longest event, minimal O2 saturation, and TST<90%. CRP decreased significantly (p< 0.001) and independently of changes in BMI (see Figs 1b and 3a, Table 5), but CRP in the final PSG in the success50% group did not differ from CRP in the non-success group (see Table D in S1 File). Again, there were no significant changes in BMI or metabolic parameters. BMI (Fig 3b) and FPG even increased. BP improved significantly (p<0.001 systolic and diastolic) and reached similar values as compared to the non-success group although the baseline values were higher in the success group (Table D in S1 File, Fig 3c).

The differences in the period of time between initial and final PSG had no influence on AHI (AHI success50%: 12.9 months [5.4–20.3]; AHI >50%: 13.3 months [6.6–22.4], p = 0.237) or on CRP (success50%: ρ = -0.011; p = 0.779; non-success: ρ = -0.010; p = 0.938).

### Results within SRBD subtypes

Defining OSAS with obstructive respiratory events comprising ≥ 50% of the total AHI, and CSAS with central respiratory events being ≥ 50% of the total AHI, we analysed the results within these two subtypes. Of the 716 initially identified patients, 606 fulfilled the criteria for OSAS, 108 those for CSAS, in two patients the predominating respiration pattern was not distinct, so these were not included in this analysis. Patients in the OSAS group yielded similar results to the overall analysis with highest CRP and BMI in the severe SRBD group and significant results with success (Tables 7 and 8).

**Table 7:** Characteristics of the OSAS group.

Total n = 606	Mild SRBD	Moderate SRBD	Severe SRBD	p-value
	(AHI≥ 5 to <15/h)	(AHI 15 to <30/h)	(AHI ≥30/h)	
n	155 (25%)	181 (30%)	270 (45%)	
CRP	2.0 [1.0–4.5]	2.7 [1.3–5.3]	4.0 [2.0–7.3]	**<0.001** severe vs moderate
				**<0.001** severe vs mild
				**0.07** moderate vs mild
Art. Hypertension yes	113 (23.2%)	146 (29.9%)	229 (46.9%)	0.01(yes vs no)
Art. Hypertension no	41 (36.0%)	34 (29.8%)	39 (34.2%)	0.01(yes vs no)
BMI	29.8 [26.4–33.5]	30.8 [27.5–35.1]	34.7 [31.1–39.3]	**<0.001** severe vs moderate
				**<0.001** severe vs mild
				**0.02** moderate vs mild

**Table 8:** Changes in CRP, QoL, systolic and diastolic blood pressure and BMI in the OSAS group for successfully treated patients.

	Success AHI<5/h	Success50%
CRP	p<0.001	p<0.001
QoL	p<0.001	p<0.001
BPsyst	p<0.001	p<0.001
BPdiast	p<0.001	p<0.001
BMI	p<0.001	p<0.001

In CSAS, the initial distribution of mild, moderate, and severe SRBD was significantly different to the one in OSAS (p = 0.01) with more severe SRBD in CSAS compared to OSAS (59% vs. 45%). CRP values were without significant differences within the severity groups, and results concerning CPR and BMI with SRBD treatment lacked statistical significance, however, quality of life as well as blood pressure values yielded similar improvement as in the overall analysis (Tables 9 and 10).

**Table 9:** Characteristics in the CSAS group.

Total n = 108	Mild SRBD	Moderate SRBD	Severe SRBD	p-value
	(AHI≥ 5 to <15/h)	(AHI 15 to <30/h)	(AHI ≥30/h)	
N	16 (15%)	28 (26%)	64 (59%)	
CRP	1.1 [1.0–2.3]	1.8 [1.0–3.5]	2.0 [1.0–5.2]	**0.054** severe vs moderate
				**0.108** severe vs mild
				**0.259** moderate vs mild
Art. Hypertension yes	9 (10.3%)	21 (24.1%)	57 (65.5%)	0.008(yes vs no)
Art. Hypertension no	7 (33.3%)	7 (33.3%)	7 (33.3%)	0.008(yes vs no)
BMI	28.1 [27.4–29.7]	28.3 [26.4–30.9]	31.7 [27.8–36.2]	**0.002** severe vs moderate
				**0.01** severe vs mild
				**0.81** moderate vs mild

**Table 10:** Changes of CRP, Qol, systolic and diastolic blood pressure and BMI in the CSAS group for successfully treated patients.

	Success AHI<5/h	Success50%
CRP	p = 0.941	p = 0.979
QoL	p = 0.029	p = 0.053
BPsyst	p = 0.020	p = 0.007
BPdiast	p<0.001	p<0.001
BMI	p = 0.687	p = 0.708

### Results with regard to type of treatment

Analysis with regard to treatment with CPAP/APAP yielded similar results to the overall analysis with respect to improvement of CRP, quality of life, blood pressure values, and increasing BMI. Significance of CRP baseline levels comparing the various severity groups was comparable to the overall results as well. Differences in prevalence of blood pressure were less distinct. In the BPAP and ASV groups, the results did not follow the trend as found in CPAP/APAP and overall (Tables 11–16). However, in BPAP, diastolic blood pressure improved in the 50%success group as did quality of life, the latter just missing statistical significance. In ASV, quality of life and blood pressure improved significantly, but not CRP. Quality of life improved more distinctly in the group reaching AHI<5/h than in the 50%success group.

**Table 11:** Characteristics in the CPAP/APAP group.

Total n = 407	Mild SRBD	Moderate SRBD	Severe SRBD	p-value
	(AHI≥ 5 to <15/h)	(AHI 15 to <30/h)	(AHI ≥30/h)	
n	81 (20%)	127 (31%)	199 (49%)	
CRP	2.0 [1.1–4.2]	2.5 [1.1–5.3]	3.4 [1.7–6.6]	**0.004** severe vs moderate
				**<0.001** severe vs mild
				**0.299** moderate vs mild
Art. Hypertension yes	64 (19.3%)	102 (30.8%)	165 (49.8%)	0.648(yes vs no)
Art. Hypertension no	16 (21.9%)	25 (34.2%)	32 (43.8%)	0.648(yes vs no)
BMI	30.2 [27.7–33.5]	30.8 [27.4–35.1]	34.7 [31.1–38.8]	**<0.001** severe vs moderate
				**<0.001** severe vs mild
				**0.455** moderate vs mild

**Table 12:** Changes of CRP, QoL, systolic and diastolic blood pressure and BMI in the CPAP/APAP group for successfully treated patients.

	Success AHI<5/h	Success50%
CRP	p = 0.007	p = 0.011
QoL	p<0.001	p = 0.001
BPsyst	p<0.001	p<0.001
BPdiast	p<0.001	p<0.001
BMI	p<0.001	p<0.001

**Table 13:** Characteristics in the BPAP group.

Total n = 32	Mild SRBD	Moderate SRBD	Severe SRBD	p-value
	(AHI≥ 5 to <15/h)	(AHI 15 to <30/h)	(AHI ≥30/h)	
n	6 (19%)	6 (19%)	20 (62%)	
CRP	2.9 [2.0–4.5]	6.0 [2.1–9.4]	6.2 [2.9–9.4]	**0.976** severe vs moderate
				**0.219** severe vs mild
				**0.485** moderate vs mild
Art. Hypertension yes	5 (17.2%)	6 (20.7%)	18 (62.1%)	0.605 (yes vs no)
Art. Hypertension no	1 (33.3%)	0 (0%)	2 (66.7%)	0.605 (yes vs no)
BMI	29.2 [25.6–33.5]	40.3 [34.6–43.4]	39.7 [33.3–44.6]	**1.000** severe vs moderate
				**0.013** severe vs mild
				**0.041** moderate vs mild

**Table 14:** Changes of CRP, QoL, systolic and diastolic blood pressure and BMI in the BPAP group for successfully treated patients.

	Success AHI<5/h	Success50%
CRP	p = 0.538	p = 0.161
QoL	p = 0.320	p = 0.058
BPsyst	p = 0.936	p = 0.657
BPdiast	p = 0.083	p = 0.012
BMI	p = 0.165	p = 0.178

**Table 15:** Characteristics in the ASV group.

Total n = 148	Mild SRBD	Moderate SRBD	Severe SRBD	p-value
	(AHI≥ 5 to <15/h)	(AHI 15 to <30/h)	(AHI ≥30/h)	
n	12 (8%)	38 (26%)	98 (66%)	
CRP	2.3 [1.7–5.4]	2.1 [1.1–4.6]	3.8 [1.7–6.9]	**0.042** severe vs moderate
				**0.407** severe vs mild
				**0.682** moderate vs mild
Art. Hypertension yes	10 (7.8%)	30 (23.3%)	89 (69.0%)	0.164(yes vs no)
Art. Hypertension no	2 (10.5%)	8 (42.1%)	9 (47.4%)	0.164(yes vs no)
BMI	31.0 [27.4–33.6]	30.4 [27.1–34.1]	33.3 [29.4–37.3]	**0.026** severe vs moderate
				**0.176** severe vs mild
				**0.946** moderate vs mild

**Table 16:** Changes of CRP, QoL, systolic and diastolic blood pressure and BMI in the ASV group for successfully treated patients.

	Success AHI<5/h	Success50%
CRP	p = 0.326	p = 0.072
QoL	p = 0.022	p = 0.004
BPsyst	p = 0.027	p = 0.004
BPdiast	p<0.001	p<0.001
BMI	p = 0.017	p = 0.017

### Results according to gender

Of all 716 patients, 512 were male (71.5%) and 204 female (28.5%). Most male patients had severe SRBD (50%), whereas only 22.9% had mild and 27.1% moderate SRBD, respectively.

Most female patients also had severe SRBD (46.9%), 26.5% had mild, and 29.2% moderate SRBD. The distribution differed significantly between male and female (p = 0.030).

Similar to the initial distribution with more male than female patients at baseline, the gender distribution according to treatment effect showed a higher percentage of male patients in all groups. However, treatment effects did not show statistically significant differences within the sexes (Table 17) with p = 0.597 in the group with final AHI<5/h and p = 0.342 in the 50%success group, respectively.

**Table 17:** Differences in gender within the groups of treatment effect.

Treatment effect	Male	Female
AHI <5/h	73%	27%
AHI ≥ 5/h	71%	29%
Success50%	72%	28%
nonSuccess50%	67%	33%

Initial and final CRP was higher in female patients. However, change was similar in female and male patients (Table 18).

**Table 18:** CRP within gender.

	Male	Female	p
Initial CRP	2.5 [1.2–5.2]	3.8 [1.7–6.9]	0.001
Final CRP	2.4 [1.1–4.6]	2.6 [1.5–5.9]	0.008
Change of CRP	-0.2 [-1.4–.8]	-0.2 [-2.0–.65]	0.656

### Results with respect to medication and its changes

We analysed some major medication groups with possible influence on respiration, metabolic and cardiovascular parameters. Antihypertensive drugs and antidepressants comprised the highest percentage of medication used (Table 19). Significant changes occurred within groups of antihypertensive medication as well as in opioids, although the latter did not comprise a large group neither in the initial nor in the final examination (Table 20). Patients with continuous medication, those without, and those with changes (either medication added or removed) in the analysed medication groups had different results in CRP, systolic and diastolic blood pressure (Table 21). Interestingly, in a post hoc analysis of significant medication, angiotensin-receptor-blockers only influenced CRP, but not blood pressure parameters. However, this effect was apparent only in comparison of newly added ARB to no usage at all. Continuous medication with this substance did not lead to differences in comparison to no usage nor in comparison to newly added ARB (Table 22). ARB usage did not influence success rates, whereas betablockers, antidepressants, and central antihypertensives did (Table 23). Comparing the successful treatment group (AHI<5/h) with the non-successful group (AHI≥5/h), a higher percentage of patients with AHI<5/h never had betablockers (55.9% vs 48.7%) or had additional betablockers at the final PSG (6.6% vs 5.5%). Interestingly, in the success groups, a higher percentage of patients never had antidepressants (AHI<5/h: 77.9%) nor central antihypertensives (success50%: 93.2%).

**Table 19:** Number of patients receiving medication.

Medication	Initial PSG	Final PSG
Betablockers	284 (40%)	302 (42%)
ACE-inhibitors	235 (33%)	255 (36%)
Angiotensin-receptor-blockers	114 (16%)	135 (19%)
Central antihypertensives	35 (5%)	37 (5%)
Antidepressants	147 (21%)	158 (22%)
Neuroleptics	33 (5%)	32 (5%)
Tranquilizer	22 (3%)	25 (4%)
Opioids	13 (2%)	23 (3%)
Antiparkinson therapy	20 (3%)	27 (4%)
Thyroid replacement therapy	124 (17%)	118 (17%)
PD5-Inhibitors	2 (0%)	3 (0%)
Endothelin-receptor-antagonists	5 (1%)	7 (1%)
Systemic Glucocorticoids	2 (0%)	4 (1%)

**Table 20:** Change of medication from initial to final PSG.

Medication	n	Initial	Final	added	removed	significance
Betablockers	711	283 (39,8%)	301 (42,3%)	45 (6%)	27 (4%)	0.044
ACE-inhibitors	710	233 (32,8%)	255 (35,9%)	47 (7%)	25 (4%)	0.013
Angiotensin-receptor-blockers	712	114 (16,0%)	135 (19,0%)	39 (5%)	18 (3%)	0.008
Central antihypertensives	708	35 (4,9%)	37 (5,2%)	17 (2%)	15 (2%)	0.860
Antidepressants	711	144 (20,3%)	158 (22,2%)	31 (4%)	17 (2%)	0.059
Neuroleptics	706	32 (4,5%)	32 (4,5%)	8 (1%)	8 (1%)	1.000
Tranquilizer	711	22 (3,1%)	25 (3,5%)	11 (2%)	8 (1%)	0.648
Opioids	706	13 (1,8%)	23 (3,3%)	12 (2%)	2 (0%)	0.013
Antiparkinson therapy	710	20 (2,8%)	27 (3,8%)	14 (2%)	7 (1%)	0.189
Thyroid replacement therapy	706	122 (17,3%)	117 (16,6%)	9 (1%)	14 (2%)	0.405
PD5-inhibitors	704	2 (0,3%)	2 (0,3%)	1 (0%)	1 (0%)	1.000
Endothelin-receptor-antagonists	699	5 (0,7%)	6 (0,9%)	1 (0%)	0 (0%)	1.000
Systemic Glucocorticoids	699	2 (0,3%)	4 (0,6%)	4 (1%)	2 (0%)	0.687

**Table 21:** Differences in CRP, QoL, systolic and diastolic blood pressure and BMI in patients with continuous/no/removed/added medication.

Medication	CRP	QoL	systolic	diastolic	BMI
Betablockers	0.871	0.952	**0.011**	0.097	0.174
ACE-inhibitors	0.495	0.418	0.175	0.299	0.197
Angiotensin-receptor-blockers	**0.025**	0.673	0.362	0.254	0.647
Central antihypertensives	0.554	0.829	0.734	0.866	0.363
Antidepressants	0.436	0.225	0.113	**0.002**	0.332

**Table 22:** Post hoc analysis of significant medication groups.

Medication	Post hoc	No vs added	Continuous vs added	No vs cont.	Cont. vs removed	No vs removed	Added vs removed
Betablockers	Systolic BP	**p<0.001**	**p = 0.009**	p = 0.497	p = 0.825	p = 0.540	p = 0.101
ARB	CRP	**p = 0.005**	p = 0.060	p = 0.191	p = 0.966	p = 0.564	p = 0.303
Antidepressants	Diastolic BP	**p = 0.020**	**p = 0.005**	p = 0.100	**p = 0.005**	**p = 0.012**	p = 0.658

**Table 23:** Medication and success.

	Success AHI<5/h	Success50%
Betablockers	**0.045**	0.353
ACE-inhibitors	0.567	0.237
Angiotensin-receptor-blockers	0.816	0.052
Central antihypertensives	0.206	**0.031**
Antidepressants	**0.039**	0.158

## Discussion

Our data are in accordance to previous studies (16, 17, 27, 28), describing an association of SRBD with inflammation. Patients with severe SRBD had the highest BMI, highest CRP and HbA1c, highest BP, and a trend towards higher prevalence of atrial fibrillation, coronary artery disease, myocardial infarction, apoplexy and renal insufficiency, according to the hypothesis that SRBD causes or interacts with cardiovascular and metabolic sequelae. Lack of statistical significance concerning the differences in co-morbidities (AF, CAD, Apo, renal insufficiency) regarding different levels of SRBD severity may be due to the fact that most patients were referred by respiratory specialists, whereas only a few were referred by cardiologists, nephrologists, and neurologists, which may skew the prevalence of associated co-morbidities.

During SRBD specific therapy, patients did not improve their BMI. Nevertheless, patients with AHI ≥30/h at baseline showed a significant reduction in CRP without changes in their metabolic parameters (BMI, HbA1c, FPG, TG, LDL, Chol). This suggests that sleep apnoea specifically caused increased CRP values which were corrected by specific therapy. This is in accordance to the previous findings by Arnardottir *et al*. [27] who concluded that CRP levels were independently associated with SRBD severity with a greater impact of obstructive sleep apnoea on inflammatory biomarkers in obese patients. Our results suggest that severity of SRBD has a greater influence on inflammatory biomarkers than weight, however, the average of all groups in our study was obese (29.2kg/m², 30.2kg/m², 34.6kg/m²). Improvement of SRBD had an impact on CRP only in patients with an initial AHI ≥30/h, even in the absence of weight loss or improvement of BMI. The findings of Punjabi &Beamer [28] support the improvement of CRP independently of BMI.

In previous studies that failed to find an obesity-independent association between SRBD and CRP [29, 30, 31], patients with severe co-morbidities were excluded. Thus, only early stages of influence of SRBD on cardiovascular diseases via inflammation may have been covered. The inflammatory impact of SRBD may be manifest and detectable only in more advanced disease, though. The same may be true for a study where unstable cardiovascular disease was an exclusion criteria and the basis of data collection was a mailed survey [32]. Both facts may have led to the exclusion of patients with more advanced diseases. This is reflected by the overall percentage of cardiovascular disease of only 5.3% in that study.

In our study, the analysis of SRBD treatment with regard to success (AHI <5/h) yielded a significant decrease of CRP along with treatment success. Results for HbA1c and FPG were controversial. However, CRP improvement occurred despite increase in BMI. This strongly supports the causal role of SRBD for systemic inflammation and the improvement of inflammatory biomarkers with SRBD therapy, independent of BMI. The influence on cardiovascular disorders is reflected by the overall and significant decrease of BP levels. These findings are in accordance to previous findings [20, 21, 33, 34, 35]. Others were not able to support these findings [36], but they used CPAP for only four weeks, which may be too short to yield beneficial effects. In our analysis, the period of time between initial and final PSG was considerably longer, but not the same for all patients. Although success rate regarding success with AHI<5/h differed with time, it did not differ with regard to 50%success. Therefore, we conclude that the difference in AHI over the given time period was still close to AHI<5/h. Nevertheless, time period had no influence on CRP, improvement of AHI yielded improvement of CRP regardless of time, given a median time period close to 12 months.

Previous studies on CRP in SRBD as comparison are outlined in Table E and Table F in S1 File [16–19, 21, 22, 27–33, 35, 37–41].

Inflammatory markers improved if success was defined as improvement of AHI of at least 50%, but without a significant difference to non-success. Apparently reduction of AHI, even markedly, without reaching the limits of normal is not sufficient to influence markers of inflammation. This suggests that only normalisation of AHI may prevent inflammatory activation.

We analysed subtypes of SRBD as well as different treatment groups. The results for OSAS as well as for CPAP/APAP are in accordance to the overall findings. However, CSAS and other treatment forms (BPAP and ASV) did not show statistical significance in all the examined parameters. But these results must be interpreted with caution, as the groups of CSAS as well as these of BPAP or ASV were considerably smaller than those of OSAS or CPAP/APAP, respectively.

Percentage of male patients was higher in the overall study population according to known prevalence in general population [2]. As a consequence, the portion of male patients remained higher in all groups. However, treatment effect did not differ between male and female patients.

The analysis of medication usage and changes yielded controversial results which should be interpreted with caution as the analysis was done post hoc and data regarding medication were not available for all patients at both time points. Not surprisingly, addition of betablockers influenced systolic blood pressure. However, changes in other antihypertensive drugs did not. Addition of antidepressants had a similar effect on diastolic blood pressure. Interestingly, a higher percentage of successfully treated SRBD patients never had antidepressants or central antihypertensives. But as the non-success groups did not have a higher percentage of these medications, a direct link to success or non-success of SRBD treatment cannot be assumed.

Addition of ARB had an impact on CRP. However, continuous treatment with ARB or their removal did not. So again, an association is difficult to establish. A multiplied effect of newly added ARB and SRBD treatment could be possible.

The only constant result in all analyses seems to be a direct link between SRBD and CRP. This offers a possibility to influence consequences of inflammation such as cardiovascular disease with SRBD treatment. Thus, despite the relationship of obesity and SRBD [42], SRBD must be addressed on its own irrespective of weight and weight changes. This must be done thoroughly, as only a tight control of SRBD with reaching normal limits of AHI seems to be able to reduce the inflammatory burden due to SRBD.

**Fig 4:**
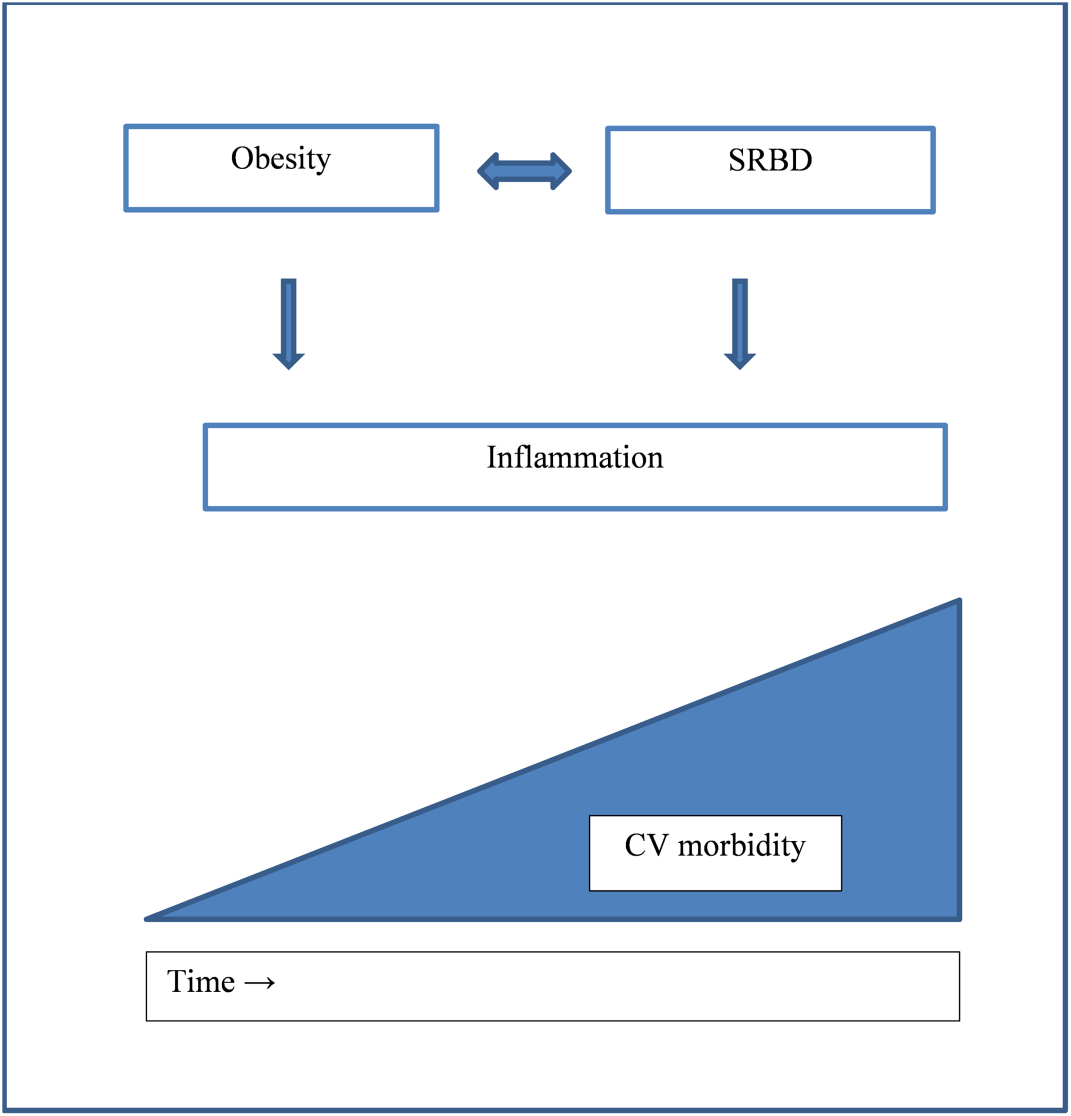
Relationship of SRBD with CRP, obesity, and cardiovascular morbidity.

The more distinct findings of association of SRBD and CRP in patients with cardiovascular co-morbidities warrant a higher awareness of SRBD and the necessity of normalization of AHI in these conditions, regarding SRBD not as a side-effect, but as the backbone of morbidity and mortality. On the other hand, our findings point out the aggravating effect of SRBD in its natural course and therefore emphasize the need for early treatment to prevent cardiovascular sequelae (Fig 4).

### Limitations

This study was conducted as a retrospective analysis of data and is therefore prone to confounding. However, the data had been prospectively collected in a comprehensive database and were based on polysomnography rather than polygraphy. We included all patients who fulfilled the inclusion criteria and all patients went through the follow-up, only few data were missing. Therefore, the bias may be rather small. We did not investigate a control group with a sham SRBD therapy and compared just patients based on their baseline SRBD severity and their response to SRBD therapy. This precludes the proof of a causal relationship between SRBD and systemic inflammation. However, we show some evidence that makes such a causal relationship appear very likely.

We do not consider the lack of stratification or exclusion of co-morbidities as a limitation but rather as strength of our study. Only this approach allowed for our findings in more severely affected patients and reflects everyday life and conditions.

We did not use hs-CRP analysis although the median CRP level was within normal limits. Employing hs-CRP might have led to more distinct values. Nevertheless, we found a statistically significant association which speaks in favour of a robust effect of SRBD on inflammation.

As non-success groups were distinctly smaller than success groups, comparability might have been impaired and significant differences between these groups may have been missed.

The same is true for the CSAS subgroups as well as BPAP and ASV subgroups.

## Conclusion

CRP is associated with SRBD independently of obesity. SRBD specific therapy (PAP in any form, positional therapy) can reduce inflammatory processes if normalisation of AHI and associated parameters is achieved. The association of CRP and SRBD is more distinct in severe SRBD and in patients with SRBD-related co-morbidities. As SRBD seems to act independently on morbidity and mortality, a higher awareness and effective treatment of SRBD is essential to prevent and stabilize or ameliorate cardiovascular morbidity.

## Supporting Information

S1 File **Table A**. Values at initial and final PSG. **Table B**. Comparison of success (AHI<5/h) and non-success group. **Table C**. Values in the non-success group at initial and final PSG. **Table D**. Comparison success (50% reduction) to non-success. **Table E**. Studies on CRP in SRBD. **Table F**. Studies on effect of CPAP.
(DOCX)
