# Supplementary material for: Sleep Related Breathing Disorders and Inflammation — The Missing Link? A Cohort Study Evaluating the Interaction of Inflammation and Sleep Related Breathing Disorders and Effects of Treatment
Source: PLoS One. 2015 Sep 10;10(9):e0137594. doi: 10.1371/journal.pone.0137594 (PMC4565554; doi:10.1371/journal.pone.0137594)
Supplement: S1 File — Table A. Values at initial and final PSG. Table B. Comparison of success (AHI<5/h) and non-success group. Table C. Values in the non-success group at initial and final PSG. Table D. Comparison success (50% reduction) to non-success. Table E. Studies on CRP in SRBD. Table F. Studies on effect of CPAP. (DOCX) [file pone.0137594.s001.docx]

Supplement

**Table S- A.** Values at initial and final PSG; median (interquartile range)

| **postAHI ≥ 5/h** |  | **initial PSG** | **final PSG** | **Change** | **sign. change** |
| --- | --- | --- | --- | --- | --- |
| Age | years | 63.3 (54.0 - 70.6) | 64.5 (55.2 - 71.7) | .9 (.4 - 2.0) | .000 |
| Height | cm | 170.0 (163.0 - 176.0) | 170.0 (163.0 - 176.0) | .0 (.0 - .0) | n/a |
| Weight | kg | 94.0 (82.0 - 105.8) | 93.0 (82.0 - 106.5) | .0 (-2.0 - 3.0) | .389 |
| BMI | kg/m² | 32.8 (29.0 - 36.6) | 33.0 (29.0 - 37.2) | .0 (-.7 - 1.0) | .344 |
| HDL | mg/dl | 49.0 (39.0 - 59.0) | 49.0 (40.0 - 57.0) | -1.0 (-5.0 - 5.0) | .759 |
| LDL | mg/dl | 109.0 (90.0 - 130.0) | 106.0 (88.0 - 130.0) | 1.0 (-17.0 - 11.0) | .661 |
| Chol | mg/dl | 187.0 (162.0 - 214.0) | 180.0 (154.0 - 211.0) | -1.0 (-18.0 - 14.0) | .325 |
| CRP | mg/l | 3.5 (1.7 - 6.9) | 3.0 (1.5 - 5.8) | -.2 (-2.3 - 1.0) | .047 |
| TG | mg/dl | 135.0 (99.0 - 187.0) | 132.0 (92.0 - 189.0) | -1.0 (-26.0 - 32.0) | .761 |
| FPG | mg/dl | 106.0 (96.0 - 123.0) | 104.0 (96.0 - 120.0) | 1.0 (-9.0 - 8.0) | .951 |
| HbA1c | IFCC: mmol/mol | 39.9 (36.6 - 44.3) | 39.9 (37.0 - 44.3) | .0 (-1.7 - 2.2) | .256 |
| AHI |  | 31.6 (19.1 - 53.2) | 9.1 (6.7 - 14.7) | -21.2 (-38.1 - -8.0) | .000 |
| Longest event | sec | 62.0 (45.8 - 92.5) | 56.9 (40.0 - 87.0) | -3.4 (-30.0 - 20.0) | .074 |
| Min O2 | % | 78.9 (69.9 - 82.8) | 85.5 (82.0 - 87.9) | 6.1 (3.0 - 14.0) | .000 |
| ODI |  | 31.5 (19.1 - 54.0) | 9.1 (6.4 - 15.5) | -19.6 (-36.5 - -7.3) | .000 |
| % TST<90 |  | 18.8 (5.8 - 47.5) | 1.2 (0.2 - 5.7) | -13.2 (-40.0 - -2.1) | .000 |
| BP syst | mmHg | 140.0 (124.0 - 153.0) | 132.0 (124.0 - 152.0) | -2.0 (-16.0 - 9.0) | .036 |
| BP diast | mmHg | 88.0 (80.0 - 98.0) | 83.0 (74.0 - 94.0) | -2.5 (-13.0 - 5.0) | .000 |

**Table S- B.** Comparison of success (AHI<5/h) and non-success group; median (interquartile range)

| **initial PSG** |  | **Success (post AHI<5/h)** | **nonSuccess (post AHI≥5/h)** | **Sign.** |
| --- | --- | --- | --- | --- |
| Age | years | 60.1 (51.7 - 69.6) | 63.3 (54.0 - 70.6) | .016 |
| Height | cm | 170.0 (164.0 - 176.0) | 170.0 (163.0 - 176.0) | .526 |
| Weight | kg | 91.0 (80.0 - 106.0) | 94.0 (82.0 - 106.0) | .285 |
| BMI | kg/m² | 31.5 (27.7 - 35.6) | 32.8 (29.0 - 36.6) | .058 |
| HDL | mg/dl | 50.0 (41.0 - 61.0) | 49.0 (39.0 - 59.0) | .143 |
| LDL | mg/dl | 114.0 (92.0 - 139.0) | 109.0 (90.0 - 130.0) | .087 |
| Chol | mg/dl | 190.0 (161.0 - 220.0) | 187.0 (162.0 - 214.0) | .380 |
| CRP | mg/l | 2.6 (1.2 - 5.5) | 3.5 (1.7 - 6.9) | .002 |
| TG | mg/dl | 124.0 (94.0 - 175.0) | 135.0 (99.0 - 187.0) | .075 |
| FPG | mg/dl | 100.0 (92.0 - 113.0) | 106.0 (96.0 - 123.0) | <.001 |
| HbA1c | IFCC: mmol/mol | 38.8 (35.5 - 43.2) | 39.9 (36.6 - 44.3) | .010 |
| AHI |  | 26.5 (14.4 - 46.1) | 31.6 (19.0 - 53.2) | .001 |
| Longest event | sec | 64.6 (47.7 - 89.8) | 62.0 (45.8 - 92.5) | .786 |
| Min O2 | % | 79.9 (73.0 - 83.9) | 78.9 (69.9 - 82.8) | .008 |
| ODI |  | 25.4 (12.8 - 45.0) | 31.5 (19.1 - 54.0) | .003 |
| % TST<90 |  | 11.0 (3.0 - 37.0) | 19.0 (6.0 - 48.0) | .000 |
| BP syst | mmHg | 141.0 (130.0 - 155.0) | 140.0 (124.0 - 153.0) | .126 |
| BP diast | mmHg | 90.0 (81.0 - 97.0) | 88.0 (80.0 - 98.0) | .450 |
| **final PSG** |  |  |  |  |
| Age | years | 61.7 (52.7 - 70.9) | 64.5 (55.2 - 71.7) | .025 |
| Height | cm | 170.0 (164.0 - 176.0) | 170.0 (163.0 - 176.0) | .530 |
| Weight | kg | 92.0 (81.0 - 105.0) | 93.0 (82.0 - 107.0) | .373 |
| BMI | kg/m² | 31.7 (28.0 - 36.3) | - 33.0 (29.0 - 37.2) | .121 |
| HDL | mg/dl | 50.0 (41.0 - 61.0) | 49.0 (40.0 - 57.0) | .069 |
| LDL | mg/dl | 114.0 (90.0 - 138.0) | 106.0 (88.0 - 130.0) | .104 |
| Chol | mg/dl | 189.0 (161.0 - 219.0) | 180.0 (154.0 - 211.0) | .141 |
| CRP | mg/l | 2.3 (1.1 - 4.6) | 3.0 (1.5 - 5.8) | .010 |
| TG | mg/dl | 126.0 (90.0 - 172.0) | 132.0 (92.0 - 189.0) | .169 |
| FPG | mg/dl | 102.0 (94.0 - 115.0) | 104.0 (96.0 - 120.0) | .034 |
| HbA1c | IFCC: mmol/mol | 38.8 (35.5 - 43.2) | 39.9 (37.0 - 44.3) | .017 |
| AHI |  | 1.2 (0.4 - 2.5) | 9.1 (6.7 - 14.7) | <.001 |
| Longest event | sec | 30.0 (19.2 - 48.1) | 56.9 (40.0 - 87.0) | <.001 |
| Min O2 | % | 90.0 (87.0 - 91.0) | 85.5 (82.0 - 87.9) | <.001 |
| ODI |  | 1.2 (0.3 - 2.7) | 9.1 (6.4 - 15.5) | <.001 |
| % TST<90 |  | 0.0 (0.0 - 0.0) | 1.0 (0.0 - 6.0) | <.001 |
| BP syst | mmHg | 135.0 (125.0 - 150.0) | 132.0 (124.0 - 152.0) | .403 |
| BP diast | mmHg | 85.0 (78.0 - 92.0) | 83.0 (74.0 - 94.0) | .511 |
| **Change** |  |  |  |  |
| Age | years | 1.1 (0.6 - 1.9) | 0.9 (0.4 - 2.0) | .035 |
| Height | cm | 0.0 (0.0 - 0.0) | 0.0 (0.0 - 0.0) | n/a |
| Weight | kg | 1.0 (-1.0 - 3.0) | 0.0 (-2.0 - 3.0) | .175 |
| BMI | kg/m² | 0.3 (-0.5 - 1.1) | 0.0 (-0.7 - 1.0) | .198 |
| HDL | mg/dl | 0.0 (-5.0 - 5.0) | -1.0 (-5.0 - 5.0) | .908 |
| LDL | mg/dl | 0.0 (-15.0 - 15.0) | 1.0 (-17.0 - 11.0) | .778 |
| Chol | mg/dl | -2.0 (-18.0 - 17.0) | -1.0 (-18.0 - 14.0) | .710 |
| CRP | mg/l | -0.2 (-1.5 - 0.6) | -0.2 (-2.3 - 1.0) | .808 |
| TG | mg/dl | 1.0 (-27.0 - 24.0) | -1.0 (-26.0 - 32.0) | .838 |
| FPG | mg/dl | 1.0 (-6.0 - 10.0) | 1.0 (-9.0 - 8.0) | .121 |
| HbA1c | IFCC: mmol/mol | 0.0 (-1.9 - 2.2) | 0.0 (-1.7 - 2.2) | .822 |
| AHI |  | -25.0 (-44.1 - -12.6) | -21.2 (-38.1 - -8.0) | .001 |
| Longest event | sec | -30.0 (-57.7 - -11.0) | -3.4 (-30.0 - 20.0) | <.001 |
| Min O2 | % | 9.1 (6.0 - 15.0) | 6.1 (3.0 - 14.0) | <.001 |
| ODI |  | -23.0 (-43.3 - -10.9) | -19.6 (-36.5 - -7.3) | .005 |
| % TST<90 |  | -9.8 (-31.5 - -1.9) | -13.2 (-40.0 - -2.1) | .232 |
| BP syst | mmHg | -5.0 (-17.0 - 8.0) | -2.0 (-16.0 - 9.0) | .184 |
| BP diast | mmHg | -4.0 (-12.5 - 4.0) | -2.5 (-13.0 - 5.0) | .403 |

**Table S- C.** Values in the non-success group at initial and final PSG; median (interquartile range)

| **non Success (50%)** |  | **initial PSG** | **final PSG** | **Change** | **sign. change** |
| --- | --- | --- | --- | --- | --- |
| Age | years | 65.8 (57.5 - 71.6) | 67.6 (58.9 - 72.5) | 1.0 (0.4 - 1.7) | <.001 |
| Height | cm | 169.0 (162.0 - 175.0) | 169.0 (162.0 - 175.0) | 0.0 (0.0 - 0.0) | n/a |
| Weight | kg | 87.0 (76.0 - 100.0) | 89.0 (78.0 - 102.0) | 0.0 (-2.0 - 3.0) | .387 |
| BMI | kg/m² | 31.2 (27.3 - 36.0) | 30.8 (27.7 - 36.1) | 0.0 (-0.6 - 1.0) | .362 |
| HDL | mg/dl | 50.0 (40.0 - 62.5) | 50.0 (40.0 - 58.0) | -1.0 (-7.0 - 6.0) | .459 |
| LDL | mg/dl | 108.0 (86.0 - 132.0) | 116.0 (91.0 - 138.0) | 3.0 (-12.0 - 15.0) | .487 |
| Chol | mg/dl | 189.0 (165.0 - 218.5) | 186.5 (157.5 - 220.5) | -4.0 (-22.0 - 21.0) | .896 |
| CRP | mg/l | 3.0 (1.3 - 6.8) | 2.5 (1.3 - 6.1) | -0.1 (-1.2 - 1.0) | .602 |
| TG | mg/dl | 135.0 (86.0 - 188.0) | 121.0 (88.5 - 170.5) | -3.0 (-26.0 - 21.0) | .414 |
| FPG | mg/dl | 105.0 (90.5 – 119.0) | 103.0 (91.0 – 121.0) | 2.0 (-6.0 – 9.0) | .261 |
| HbA1c | IFCC: mmol/mol | 41.0 (37.7 - 44.3) | 39.9 (36.6 - 44.3) | 1.0 (-1.1 - 2.2) | .092 |
| AHI |  | 16.4 (9.9 - 23.6) | 12.5 (8.6 - 24.4) | -4.0 (-7.8 - 1.7) | .006 |
| Longest event | sec | 59.4 (44.0 - 86.7) | 52.0 (39.0 - 74.6) | -2.0 (-29.0 - 12.9) | .150 |
| Min O2 | % | 80.9 (75.0 - 83.9) | 84.0 (81.9 - 86.0) | 3.1 (0.0 - 7.0) | <.001 |
| ODI |  | 14.6 (10.1 - 23.2) | 12.8 (7.1 - 19.9) | -3.5 (-7.4 - 2.5) | .019 |
| % TST<90 |  | 10.6 (3.1 - 33.7) | 4.1 (0.3 - 15.4) | -3.1 (-16.0 - 0.1) | <.001 |
| BP syst | mmHg | 134.0 (120.0 - 150.0) | 130.0 (124.0 - 145.0) | -1.0 (-19.0 - 10.0) | .478 |
| BP diast | mmHg | 86.0 (78.0 - 93.0) | 82.0 (75.0 - 90.0) | -1.0 (-10.0 - 5.0) | .096 |

**Table S- D.** Comparison success (50% reduction) to non-success; median (interquartile range)

| **initial PSG** |  | **Success (50%)** | **non Success (50%)** | **Sign.** |
| --- | --- | --- | --- | --- |
| Age | years | 60.6 (52.2 - 69.7) | 65.8 (57.5 - 71.6) | .004 |
| Height | cm | 170.0 (164.0 - 176.0) | 169.0 (162.0 - 175.0) | .201 |
| Weight | kg | 93.0 (81.0 - 106.0) | 87.0 (76.0 - 100.0) | .028 |
| BMI | kg/m² | 32.0 (28.0 - 36.2) | 31.2 (27.3 - 36.0) | .122 |
| HDL | mg/dl | 49.0 (40.0 - 60.0) | 50.0 (40.0 - 62.5) | .674 |
| LDL | mg/dl | 113.0 (92.0 - 136.0) | 108.0 (86.0 - 132.0) | .245 |
| Chol | mg/dl | 189.0 (160.5 - 218.0) | 189.0 (165.0 - 218.5) | .823 |
| CRP | mg/l | 2.9 (1.3 - 5.7) | 3.0 (1.3 - 6.8) | .566 |
| TG | mg/dl | 125.5 (96.0 - 179.0) | 135.0 (86.0 - 188.0) | .702 |
| FPG | mg/dl | 101.0 (93.0 – 116.0) | 105.0 (90.5 – 119.0) | .694 |
| HbA1c | IFCC: mmol/mol | 38.8 (35.5 - 43.2) | 41.0 (37.7 - 44.3) | .082 |
| AHI |  | 29.9 (16.6 - 49.9) | 16.4 (9.9 - 23.6) | <.001 |
| Longest event | sec | 64.7 (47.5 - 90.8) | 59.4 (44.0 - 86.7) | .006 |
| Min O2 | % | 79.0 (71.9 - 83.9) | 80.9 (75.0 - 83.9) | .037 |
| ODI |  | 28.8 (15.6 - 50.3) | 14.6 (10.1 - 23.2) | .000 |
| % TST<90 |  | 13.2 (2.9 - 41.3) | 10.6 (3.1 - 33.7) | .318 |
| BP syst | mmHg | 141.5 (130.0 - 155.0) | 134.0 (120.0 - 150.0) | .004 |
| BP diast | mmHg | 90.0 (81.0 - 98.0) | 86.0 (78.0 - 93.0) | .005 |
| **final PSG** |  |  |  |  |
| Age | years | 62.1 (53.2 - 71.1) | 67.6 (58.9 - 72.5) | .006 |
| Height | cm | 170.0 (164.0 - 176.0) | 169.0 (162.0 - 175.0) | .202 |
| Weight | kg | 93.0 (82.0 - 106.0) | 89.0 (78.0 - 102.0) | .037 |
| BMI | kg/m² | 32.2 (28.4 - 36.8) | 30.8 (27.7 - 36.1) | .124 |
| HDL | mg/dl | 50.0 (41.0 - 60.0) | 50.0 (40.0 - 58.0) | .716 |
| LDL | mg/dl | 112.0 (90.0 - 136.0) | 116.0 (91.0 - 138.0) | .888 |
| Chol | mg/dl | 187.0 (159.0 - 217.0) | 186.5 (157.5 - 220.5) | .938 |
| CRP | mg/l | 2.5 (1.2 - 4.9) | 2.5 (1.3 - 6.1) | .418 |
| TG | mg/dl | 128.0 (91.0 - 181.0) | 121.0 (88.5 - 170.5) | .586 |
| FPG | mg/dl | 102.0 (95.0 – 116.0) | 103.0 (91.0 – 121.0) | .846 |
| HbA1c | IFCC: mmol/mol | 39.0 (35.5 - 44.0) | 39.9 (36.6 - 44.3) | .265 |
| AHI |  | 1.8 (0.6 - 4.2) | 12.5 (8.6 - 24.4) | <.001 |
| Longest event | sec | 35.0 (23.0 - 57.0) | 52.0 (39.0 - 74.6) | <.001 |
| Min O2 | % | 89.0 (86.0 - 91.0) | 84.0 (81.9 - 86.0) | <.001 |
| ODI |  | 1.8 (0.5 - 5.0) | 12.8 (7.1 - 19.9) | <.001 |
| % TST<90 |  | 0.1 (0.0 - 0.9) | 4.1 (0.3 - 15.4) | <.001 |
| BP syst | mmHg | 135.0 (125.0 - 151.0) | 130.0 (124.0 - 145.0) | .177 |
| BP diast | mmHg | 85.0 (77.0 - 93.0) | 82.0 (75.0 - 90.0) | .080 |
| **Change** |  |  |  |  |
| Age | years | 1.1 (0.6 - 1.9) | 1.0 (0.4 - 1.7) | .152 |
| Height | cm | 0.0 (0.0 - 0.0) | 0.0 (0.0 - 0.0) | n/a |
| Weight | kg | 0.0 (-2.0 - 3.0) | 0.0 (-2.0 - 3.0) | .856 |
| BMI | kg/m² | 0.0 (-0.6 - 1.1) | 0.0 (-0.6 - 1.0) | .615 |
| HDL | mg/dl | 0.0 (-5.0 - 5.0) | -1.0 (-7.0 - 6.0) | .248 |
| LDL | mg/dl | 0.0 (-16.0 - 14.0) | 3.0 (-12.0 - 15.0) | .543 |
| Chol | mg/dl | -2.0 (-18.0 - 16.0) | -4.0 (-22.0 - 21.0) | .766 |
| CRP | mg/l | -0.2 (-1.5 - 0.7) | -0.1 (-1.2 - 1.0) | .431 |
| TG | mg/dl | 1.0 (-27.0 - 28.0) | -3.0 (-26.0 - 21.0) | .275 |
| FPG | mg/dl | 1.0 (-7.0 – 9.0) | 2.0 (-6.0 – 9.0) | .695 |
| HbA1c | IFCC: mmol/mol | 0.0 (-2.1 - 2.2) | 1.0 (-1.1 - 2.2) | .179 |
| AHI |  | -26.6 (-45.1 - -14.2) | -4.0 (-7.8 - 1.7) | <.001 |
| Longest event | sec | -27.0 (-52.3 - -3.8) | -2.0 (-29.0 - 12.9) | <.001 |
| Min O2 | % | 9.1 (5.1 - 15.1) | 3.1 (0.0 - 7.0) | <.001 |
| ODI |  | -25.2 (-43.3 - -12.6) | -3.5 (-7.4 - 2.5) | <.001 |
| % TST<90 |  | -11.9 (-36.8 - -2.5) | -3.1 (-16.0 - 0.1) | <.001 |
| BP syst | mmHg | -4.0 (-16.0 - 8.0) | -1.0 (-19.0 - 10.0) | .118 |
| BP diast | mmHg | -4.0 (-13.0 - 4.0) | -1.0 (-10.0 - 5.0) | .123 |

**Table S- E.**: Studies on CRP in SRBD

| Study | N | Design | Outcome | Limitations | Comments |
| --- | --- | --- | --- | --- | --- |
| Arnardottir *et al* 2012 (27) | 454 | Cross-sectional cohort | OSAS severity associated with IL-6, CRP | -males > females  -no mild OSAS | Influence of OSA on CRP only in obese pts |
| Boudjeltia *et al* 2006 (37) | 49 | Cross-sectional | hsCRP independently correlates with AHI | -small sample size  -exclusion of co-morbidities | Duration of exposure to SRBD suggested as important for elevation of CRP |
| Can *et al* 2006 (38) | 62 | Observational | -CRP elevated in OSAS  -correlation CRP/AHI  -BMI same in AHI>5/h and <5AHI/h | -only male  -small sample size  -exclusion of co-morbidities | Group2 of OSAS = AHI <5/h, n=30; control group = AHI<1/h |
| Firat Guven *et al* 2012 (16) | 76 | Observational | OSA leads to elevated hsCRP | -small sample size  -co-morbidities excluded | hsCRP higher in obese pts |
| Guilleminault *et al* 2004 (29) | 156 | Cross-sectional | CRP correlates with BMI | -no morbidly obese pts  -no co-morbidities | No correlation to RDI, SaO2 after multiple regression |
| Hayashi *et al* 2006 (39) | 60 | Observational | hsCRP higher in OSAS  -correlation with hypoxia, AHI  -correlation with BMI partially | -co-morbidities excluded  -only males  -small sample size | hsCRP elevated also in non-obese OSAS |
| Kokturk *et al* 2005 (40) | 173/ 94 no OSAS | Observational | CRP higher in OSAS, and with cardiovasc. co-morbidities | -only males  -small sample size | CV group and without analysed separately |
| Lui *et al* 2009 (17) | 111 | Observational | hsCRP levels associated with OSA independent of visceral obesity | -only men  -no severe co-morbidities  -no morbidly obese pts | Visceral fat quantified by MRI |
| Punjabi & Beamer 2007 (28) | 69 | Cross-sectional | CRP is associated with SRBD independent of obesity | -small sample size  -only men  -no significant co-morbidities | CRP levels higher in moderate and severe SRBD |
| Ryan *et al* 2007 (30) | 110 | Observational | CRP not independently associated with OSA severity, but with obesity | -only men  -no co-morbidities | CPAP did not alter CPR levels, but CPAP only for 6 weeks |
| Saletu *et al* 2006 (41) | 147/ 44 no OSAS | Observational | hsCRP correlated with respiratory parameters, not independently | -no co-morbidities | -Focus on intima-media thickness  -group size not equal |
| Schiza *et al* 2010 (21) | 528 | Observational | -hsCRP associated with OSA severity independently of BMI  -CPAP reduced hsCRP | -co-morbidities excluded | -Follow-up 3/6/12 months  -compliance vs non-compliance |
| Shamsuzzaman *et al* 2002 (18) | 22 | Observational | CRP levels associated with OSA severity | -small sample size  -no significant co-morbidities | BMI- and age-matched controls |
| Su  *et al* 2013 (31) | 309 | Observational | hsCRP more strongly associated with metabolic than with OSA parameters | -cardiovascular and respiratory co-morbidities excluded | Cave: only Chinese population |
| Taheri *et al* 2007 (32) | 907 | Cross-sectional | CRP not independently associated with AHI | -unstable cardiopulmonary disease excluded | Wisconsin Sleep Cohort Study |
| Yao *et al* 2006 (19) | 316 | Cross-sectional | CRP associated with ODI, more pronounced in non-overweight pts | -only men  -only pulse oxymetry | Cave: only Japanese population |

**Table S-F**. Studies on effect of CPAP

| Study | N | Design | Outcome | Limitations | Comments |
| --- | --- | --- | --- | --- | --- |
| Friedman *et al* 2012 (33) | 325 | Meta-analysis | CPAP reduced CRP levels | 2 RCTs, 2 case-control-studies, 6 case-series | Analysis focused on post-treatment |
| Panoutsopoulos *et al* 2012 (35) | 38 | Observational | -CRP positively correlated with BMI, AHI, hypoxia time  -CPAP reduced CRP levels | -small sample size  -only men  -no significant co-morbidities | 3 months CPAP  -effect of CPAP covered in Friedman *et al.*  (30) |
| Yokoe *et al* 2003 (22) | 30 | Observational | -CRP higher in OSA than in obese controls  -CPAP reduced CRP | -only men  -small sample size | Co-morbidities no exclusion criteria, but scarce  -effects of CPAP covered in Friedman *et al.* (30) |
